# Study on Axial Compression Behavior of Concrete Short Columns Confined by Flax/Glass Fiber Hybrid-Reinforced Epoxy Resin Composites

**DOI:** 10.3390/polym14030517

**Published:** 2022-01-27

**Authors:** Lanjie Yang, Hongguang Wang, Shansong Gao

**Affiliations:** 1 China National Chemical Communications Construction Group Co., Ltd., Jinan 250102, China; yanglanjie@zhxjj.com.cn; 2 Shandong Highway Engineering Technology Research Center Co., Ltd., Jinan 250102, China; 3 School of Civil Engineering, Northeast Forestry University, Hexing Road No. 26, Xiangfang District, Harbin 150040, China; 2021115994@nefu.edu.cn

**Keywords:** hybrid FRP composites, short concrete columns, axial compression, ultimate bearing capacity

## Abstract

In this study, we aimed to explore the effect of concrete short columns confined by flax/glass fiber hybrid-reinforced epoxy resin (FFRP/GFRP) composites. Taking the same fiber hybrid ratio and different paving orders as parameters, analysis of the axial compressive mechanical properties of eight groups of FFRP/GFRP composite-confined concrete short columns, including one group of flax fiber-reinforced epoxy resin (FFRP) composite-confined concrete short columns and one group of unconstrained concrete short column, was conducted. The effects of different layering sequences on failure modes, load–displacement curves, energy dissipation ductility and the stress–strain relationship of hybrid composite-confined concrete short columns were analyzed. The results show that the axial compression failure modes of FFRP/GFRP composite-confined concrete short columns with the same hybrid ratio and different paving sequences were basically the same, and the CC-H6 group was the most prominent. The ultimate bearing capacity and axial deflection were 91.05% and 11.49% higher than those of the control group (CC-FFRP), and the energy dissipation coefficient was also the largest, at 9.79. The failure trend of the stress–strain curve of the confined concrete short column specimens was basically the same, and the stress and axial strain of the members were increased by 247.9~292.5% and 486.7~701.0%, respectively.

## 1. Introduction

Synthetic fiber-reinforced polymer composites have excellent mechanical properties, including a glass fiber tensile strength of 3400~3700 MPa and a carbon fiber tensile strength of 3500~4500 MPa [1,2,3,4], and have been widely used in the field of civil engineering reinforcement. The tensile strength of fiber-reinforced plastic (FRP) composites is several times higher than that of ordinary steel bars, and it also has good corrosion resistance and can be processed into various shapes according to the actual engineering needs [5,6]. Carbon fiber composite materials and glass fiber composite materials are the most widely used to restrain concrete columns, beams and plates and repair damaged concrete structures, which have achieved remarkable results [7,8,9,10]. However, these synthetic fibers also have some disadvantages, such as high price and non-recyclability, which make them neither economic nor conducive to environmental protection and sustainable development [11,12]. In addition, the energy consumed in the manufacturing process of synthetic fibers is not renewable, and a certain amount of carbon dioxide is released, which also causes environmental problems [13].

In recent years, composite materials consisting of natural plant fibers, such as flax, jute, ramie, hemp, etc., have attracted the attention of researchers in various fields. They have the advantages of being lightweight, high-strength, degradable, economic, practical, and providing environmental protection, and they meet the needs of the current international development trend [14,15,16]. Among these composites, the tensile strength and stiffness of flax fiber can be up to 1500 MPa and 90 GPa, respectively, with low density (1.5 g/cm^3^) and good low-cost characteristics [17,18,19]. Compared with synthetic fibers (carbon fiber, glass fiber, etc.), the main disadvantage of plant fiber-reinforced composites is that their mechanical properties arse relatively weak, which limits their application range. The reasonable mixing of plant fiber and artificial synthetic fibers enhances the resin composite material, thus making up for the shortcomings of plant fibers and artificial synthetic fibers, so as to achieve a good effect that cannot be achieved by a single type of fiber and achieve a balance between cost and performance [20,21].

Nauman Wahab et al. employed a jute–polyester hybrid fiber (JFRP) composite material to reinforce a concrete cylinder for an axial compression test [22]. The results show that the compressive strength, strain and ductility index of the reinforced specimens were 1.24~2.61 times, 1.38~8.97 times and 4.94~26.5 times higher than those of the unreinforced specimens, respectively. JFRP composites could be used in concrete structures with low requirements for strength and ductility, and could also be used to prevent spalling of the concrete protective layer and water infiltration. Yuanyuan Xia et al.’s specimens were made by casting concrete into flax/basalt mixed fiber tubes prepared by the winding process to study the composite compressive properties [23]. The results show that the fiber hybrid mode of HFRP affects the failure mode of the confined concrete column, and the ultimate strength and strain of the HFRP tube-confined concrete with B1F4B1 and B1F8B1 were the best, which was similar to the tensile test results of HFRP sheet.

In this experiment, flax fibers were used as the plant fibers, which have a low price and high specific strength, while glass fibers were used as the synthetic fibers. Using the hand paste method to prepare the flax/glass fiber hybrid-reinforced epoxy resin (FFRP/GFRP) composites with the same hybrid ratio, different layer sequences were used to observe how they affect the performance of confined concrete short columns with regard to axial compression, the failure modes of the axial compression performance test, load–displacement curves, ductility and the energy stress–strain relationship. This study could provide a reference for practical engineering applications and help to meet the performance and strength requirements of different structures in practical applications.

## 2. Materials and Methods

### 2.1. Test Materials and Equipments

Bidirectional flax fiber twisted yarn cloth (surface density of 240 g/m^2^ and volume density of 1.5 g/cm^3^) was purchased from Harbin Flax Textile Co., Ltd., Harbin, China. Bidirectional glass fiber no-twist gauze (EWR400E-100, surface density is 400 g/m^2^, volume density is 2.5 g/cm^3^) was purchased from China Jushi Group Co., Ltd., Jiaxing, China.

Epoxy-impregnated A and B adhesives (MH-301), for which the density is 1.2 g/cm^3^, were provided from Shanghai Miaohan Construction Technology Co., Ltd., Shanghai, China. The mechanical properties of pure epoxy resin were tested according to ASTM D790 (standard test method for the flexural properties of unreinforced and reinforced plastics and electrical insulating materials) and ASTM D2344/D2344M (standard test method for the short beam strength of polymer matrix composite materials and their laminates), as shown in Table 1.

In this paper, the Portland cement (P.O. 42.5) was produced by Harbin Cement Co., Ltd., Harbin, China. The fine aggregate was selected as natural river sand in Harbin City, with particle sizes less than 4.75 mm and good grading. The coarse aggregate was selected as 10~20 mm basalt gravel, and the water reducer used FND naphthalene water reducer, with a water reduction rate of 22%.

Table 2 shows the main test instruments and equipment used in the preparation of FFRP/GFRP composites and the axial compression performance test of the confined concrete cylinder.

### 2.2. Preparation of Composite-Confined Concrete Short Column

**Figure 1:**
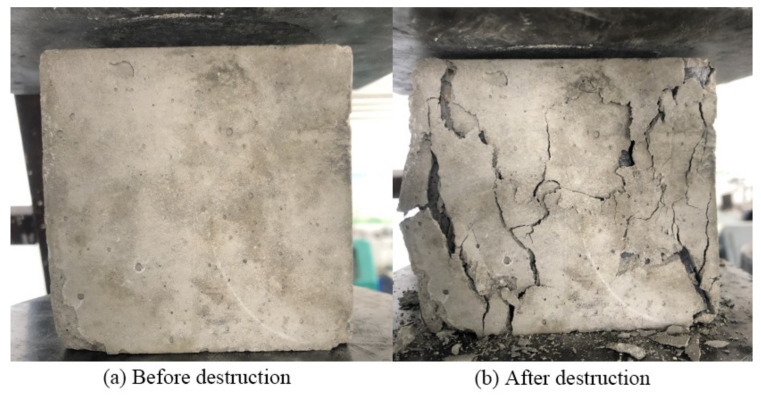
Unconfined compression test of cube.

The casting mold adopted the electric flux circular test mold with a diameter of 100 mm and a height of 200 mm. The designed strength of the concrete was C30, and the appropriate mix ratio was selected according to the design specification of the ordinary concrete mix ratio (as shown in Table 3). In order to facilitate the later demolding, before pouring the concrete, the inside of the mold was coated with lubricating oil. When pouring, we first poured half of the volume of the concrete into the mold, and put the mold on the vibration table to vibrate it fully; then, we poured in the rest of the concrete until the mold was full, with full vibration until the concrete was uniform, and finally used a shovel to remove the excess concrete and wipe smooth. Starting from the end of pouring, demolding occurred 24 h later. The demolded specimens were maintained for 28 days. According to the test provisions in the test method for the mechanical properties of ordinary concrete (GB/T 50081-2011), the average unconfined compressive strength was measured to be 36.23 MPa (as shown in Figure 1).

In order to increase the reliability of the test results, the test was divided into nine experimental groups and one control group, and each experimental group included 30 specimens of three same types. The test schedule was shown in Table 4.

**Figure 2:**
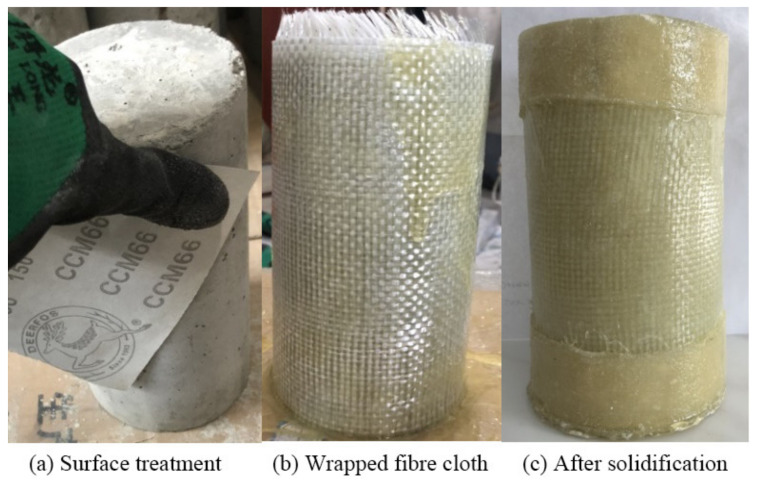
Fabrication process of composite-confined concrete short column.

The wrapping method was layered—that is, after each layer of epoxy resin was administered, a layer of fiber cloth was wrapped around the column, and this step was repeated a total of eight times. A quarter of the circumference (80 mm) was taken as the lap length, and the lap position of each layer could not overlap. In the test, due to the weak end constraint, three layers of flax fibers with a width of 4 cm were affixed to the upper and lower ends of the component in the test. The operation steps of the restraint wrapped specimen were as follows:

(1) As shown in Figure 2a, after the curing age reached 28 days, the concrete cylindrical specimen was taken out from the standard curing chamber. Sandpaper was used to polish the surface of the specimen. After blowing the floating ash, an acetone solution was used to scrub the surface.
(2) The flax fiber sheet was cut to a certain size and put into the ultrasonic cleaning machine (distilled water, room temperature) for reaction for 40 min to remove impurities, and then was taken out and dried for later use. The flax fiber and glass fiber sheet was cut to a size of 400 mm × 300 mm and put into the oven. The drying treatment was conducted at a constant temperature of 60 °C until a constant weight was attained, and then the sample quality was measured, and data were recorded;
(3) We mixed the composite according to the mass proportion 2:1, configured group A and group B with epoxy resin impregnation glue, and used a glass rod to stir the impregnation glue until the color distribution was uniform. Then, the impregnation glue was coated on the surface of the specimen, and the fiber sheet was wound around the concrete cylinder in the direction of fibers several times, and the internal bubbles were extruded. We repeated the above steps to the specified number of coating layers, then coated the outermost layer with epoxy resin adhesive (as shown in Figure 2b). Finally, three flax fiber layers with a width of 4 cm were pasted at the upper and lower ends of the specimen to strengthen the ends.
(4) After curing for 7 days at room temperature, the upper and lower parts of all specimens were leveled. If the end of the specimen was uneven, the stress concentration phenomenon occurred under the action of axial compressive stress, leading to the specimen breakage in advance. The end leveling was mainly made of high-strength gypsum. The high-strength gypsum added with an appropriate amount of water was stirred evenly and coated on the end of the specimen. The gypsum was compacted with a flat slide plate and the upper and lower ends were made sufficiently level, as shown in Figure 2c.
(5) Finally, we determined the paste position of the test strain gauge and polished it smooth for later use.

### 2.3. Experimental Method

**Figure 3:**
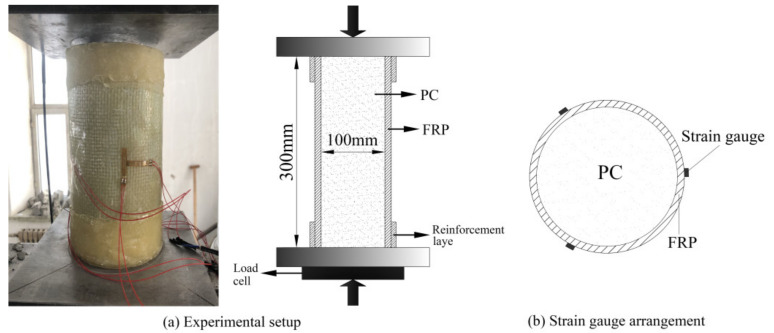
Loading device and strain gauge.

The maximum compression force of the hydraulic testing machine used in this test was 500 t, and the average compression rate was 0.5 MPa/s. The ultimate bearing capacity and axial/circumferential strain data were collected. The test point layout of the specimen is shown in Figure 3. Three longitudinal and three circumferential strain gauges were arranged at equal intervals around the circumference of the specimen. In order to facilitate the collection of longitudinal deformation and circumferential strain of the specimen during compression, a strain gauge was attached to the same wrapping material as temperature compensation. The resistance strain gauge used was 20 mm × 3 mm in size consisted of a glue base. The paste diagram is shown in Figure 3.

### 2.4. Measures to Control Test Errors

The control in the test process was also very crucial, which was an important factor related to the quality of the test data. Therefore, a series of measures were taken in this test to ensure the accuracy and reliability of the test data.

The following measures were taken to reduce the errors in the test:

(1) After wrapping the fiber around the concrete cylinder, the surface of the cylinder was leveled with a plastic scraper, and the composite material was uniformly wrapped in the concrete cylinder, so that the compression of the cross-section was uniform.
(2) We adopted the method of laser wiring to determine the specific sticking position of strain gauge to ensure it was horizontal and vertical. It was then carefully polished with fine sandpaper until smooth.
(3) Special glue was used for strain gauge bonding to better force the strain gauge and material together, prevent the risk of damage in advance, and record more accurate and effective data.
(4) In order to ensure that the specimen was subjected to uniform load and axial compression, the upper and lower ends of the specimen were made of high-strength gypsum as the leveling layer, so as to avoid local stress of the specimen.
(5) Before the test, the pressure sensor was calibrated and the strain gauge was checked again to check that it was intact.

## 3. Results and Discussion

### 3.1. Failure Mode and Process

**Figure 4:**
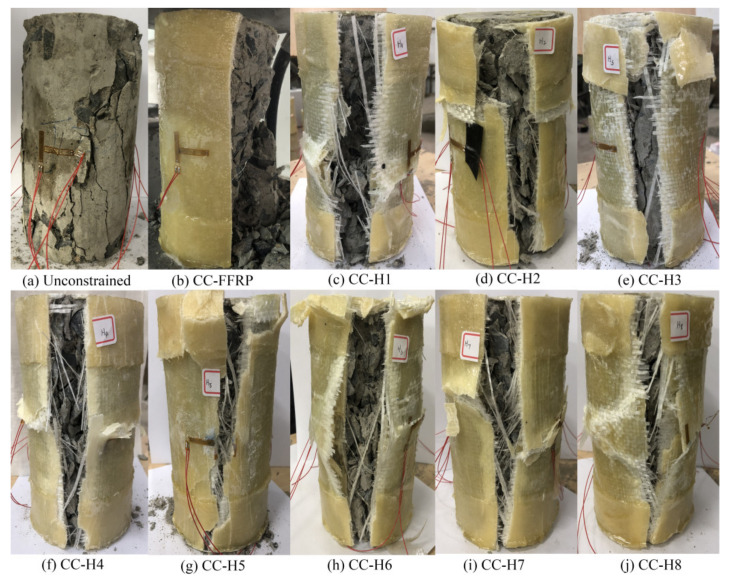
Failure modes of concrete columns under unconstrained and different constraint conditions under axial compression.

The failure mode of the specimen is shown in Figure 4a. For the unrestrained concrete column, there was no obvious damage phenomenon on the surface of the concrete column due to the small axial load at the initial stage of loading. When the axial load increased to about 40% of the peak load, several parallel vertical micro-cracks appeared on the upper surface of the cylinder. When the peak load increased to about 80%, the original cracks widened significantly, the number of new cracks increased rapidly, and each crack gradually extended and expanded to the lower part, accompanied by a subtle cracking sound. When the axial load reached the peak value, the vertical main crack of the concrete column rapidly penetrated the full height of the column, and the concrete block split by the column fell off, accompanied by an obvious bursting sound. The bearing capacity of the specimen plummeted, showing significant brittle failure characteristics.

The concrete cylinder restrained by the pure flax fiber sheet is in the elastic stage at the initial loading stage. The mechanical behavior of the whole specimen is similar to that of the unrestrained concrete cylinder, and the surface of the specimen does not change significantly. As the load continues to increase, the binding effect of fiber cloth is gradually revealed, the toroidal volume of specimens begins to expand, and the toroidal tensile strain value of the FFRP composites increases rapidly. When the axial load reaches about 60% of the ultimate load, the cylinder expands and breaks in the middle, the internal concrete strength increases over the limit, some of the fibers fracture, along with the load increasing, and the thrum becomes larger and more and more intensive, until the specimen, with a crackling sound, is destroyed, and the ultimate bearing capacity peaks, releasing energy. The failure mode is cracking in the middle of the cylinder first, and then spreading to both ends. Most of FFRP composite materials and concrete fragments splash, which is dangerous to a certain extent. A large number of concrete fragments scatter around the test bed, and the core concrete is seriously damaged, as shown in Figure 4b. It is found from the failure specimen that there are many concrete fragments attached to the cracked composite plate in the internal test, which indicates that the interface between the concrete and the composite plate has good occlusion performance, and the effect of co-coordination force occurs.

The failure mode of the HFRP composites (H1-H8) is shown in Figure 4c–j, and the overall failure process is basically similar. In the initial stage of loading, there is no significant change in appearance, and the core concrete changes little in this stage, as with the FFRP cloth-constrained concrete column, and the fiber cloth does not yet play a role of constraint. As the load continues to increase, the specimen volume expands, the fibers are subjected to tension, part of the resin peels off, and the sound is increased. The cylindrical volume increases obviously and the intermediate part is most serious, followed by a huge crack, far greater than that of the FFRP cloth, with the destruction of the confined concrete column, and the ultimate bearing capacity peaks. Cracking spreads from the first central crack and extends to both ends, and no obvious phenomenon of broken concrete and fiber cloth splash occurs, with only a small amount of slag concrete falling onto the test bench. After the failure of the specimen, a large fracture mouth is visible on the specimen. It can also be seen from the fracture mouth that the internal force is a breakthrough point in the middle section, and the final failure occurs. After that, the bearing capacity drops rapidly, leading to the complete withdrawal of the cylinder. The damage degree of the internal concrete is more serious than that of the concrete column restrained by FFRP composites, and the concrete fragments are smaller, but the whole concrete is in the form of a vertebral body, which indicates that HFRP composites have a strong restraining and protective effect on the core concrete.

In general, the failure process of plain concrete cylinder is mainly circumferential expansion, and vertical cracks appear on the periphery during the axial compression performance test, leading to longitudinal cracks along the concrete cylinder. Because the compressive strength of concrete material is 10 times its tensile strength, it cannot prevent the development of longitudinal cracks. The damaged concrete surface cracks, divides, and then falls off. The cracks between the concrete fragments extend in the same direction as the loading axis. Compared with the concrete cylinder without restraint treatment, the concrete cylinder wrapped with flax/glass fiber can have a better restraint effect. Using this method, the volumetric expansion and crack development of the concrete cylinder under compression conditions are limited, and the peak load and overall failure deformation of the concrete cylinder are obviously increased under the joint action of axial and peripheral wrapping forces. Because the lateral binding force of HFRP composites is fully exerted, and the upper and lower parts are strengthened, the lateral deformation of the end part is small, the deformation of the middle part is large, the fracture of the composite plate is neat, and the core concrete is in the conical failure form.

### 3.2. Characteristic Load and Load-Displacement Curve

**Figure 5:**
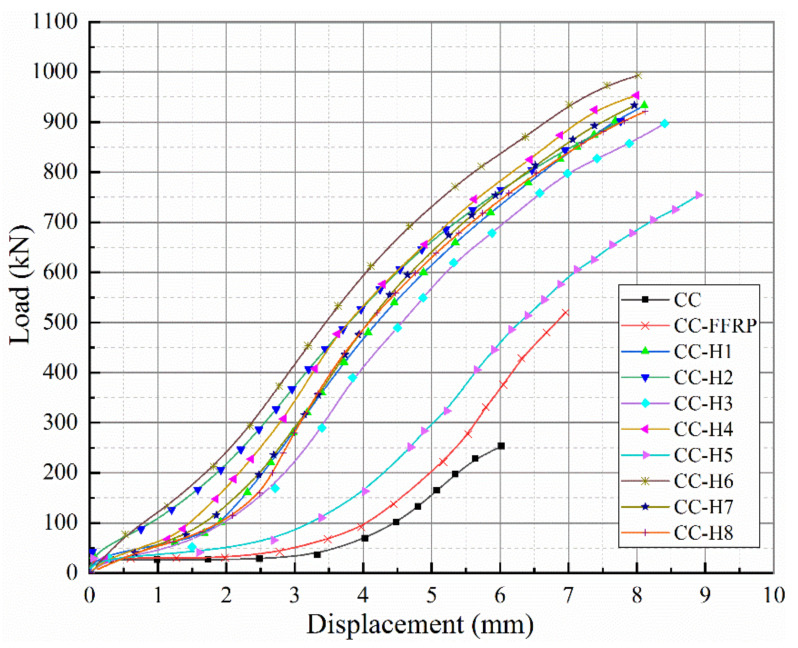
Load–displacement curves of concrete cylinders under unconstrained and diverse constraint conditions.

Figure 5 shows the load-displacement curves of plain concrete columns under various constraints for axial compression tests. The peak load is significantly increased with the restraint of fiber distribution, and the degree of increase is slightly different with different layering sequences (as shown in Table 5), but the overall loading failure trend is basically the same, which is mainly divided into the elastic change stage, the crack working stage and the critical failure stage.

Table 5 shows the comparison of the influence of each constraint mode on the ultimate cracking capacity and axial deflection of concrete cylindrical members. *P_u_* and *δ_u_* are the ultimate load and axial ultimate deflection of concrete cylindrical members under failure. Δ*P_u_* and Δ*δ_u_* are the percentage of improvement in the bearing capacity and deflection of concrete cylinders after being reinforced with fiber cloth. It can be found that the ultimate bearing capacity of the unconstrained cylinder is only 253.19 kN and the axial deflection is 6.02 mm. The bearing capacity of concrete cylinders is improved obviously by using fiber cloth to restrain them, mainly because FRP provides circular restraint, which improves the bearing capacity. When the flax/glass fiber hybrid composites were used to restrain concrete members at the same mixture ratio, CC-H6 showed the most outstanding performance. Compared with the control group (CC-FFRP), the ultimate bearing capacity of CC-H6 was increased by 91.05%, and its axial deflection was also increased by 11.49%. The ultimate bearing capacity of CC-H5 is not greatly improved, but the deflection extension is improved by 27.87%, which is similar to the conclusion regarding the mechanical properties of HFRP composites in Section 2 above. The ultimate failure load value of CC-H6 increases the most, but its axial deflection variation is the lowest compared with other HFRP composites. The slope of the load–displacement curve is the steepest, and the load lifting rate is faster. The influence of other hybrid layering sequences on the axial compressive properties of concrete columns is not significant, ranging between that observed for the CC-H6 and CC-H5 groups. This is because the axial compressive tests of concrete columns constrained by fiber composite materials mainly test the tensile properties of the constrained materials, followed by the strength of the bending properties.

Above all, the test results show that on the one hand, the flax/glass fiber hybrid composite material can effectively enhance the axial compression performance of the concrete column, the ultimate bearing capacity of the concrete column is enhanced, and the effectiveness of the structure constraint is verified, and on the other hand, the axial deflection of concrete cylinder is improved, and the deformation resistance of concrete cylinder is improved. Therefore, HFRP-reinforced concrete columns not only improve the bearing capacity, but also meet the ductility requirements of the structure.

### 3.3. Ductility Analysis of Energy Dissipation

**Figure 6:**
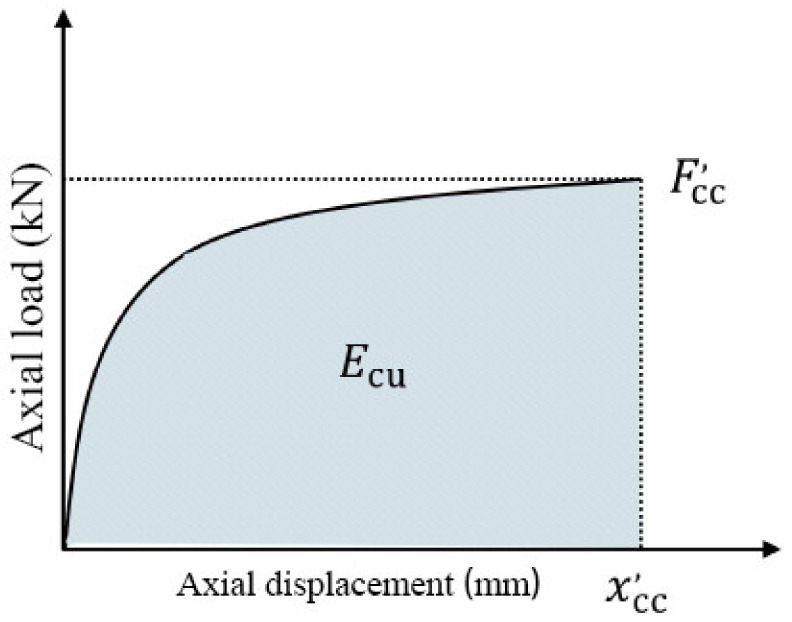
Schematic diagram of fracture energy calculation.

Ductility is mainly determined by the amount of energy absorbed by the concrete member during the stress process. The less energy is absorbed, the worse the ductility and the weaker the deformation resistance. In order to further explore the influence of the constraint on energy dissipation capacity, this chapter introduces *E_cu_* to describe the change in the specimen in the process of absorbing stress energy, as shown in Figure 6.

Its value is the area of the curve formed by the load–displacement curve of axial compression and the horizontal axis—that is, the integral solution of the curve is carried out from zero axial displacement to the maximum deflection displacement. The energy dissipation coefficient *λ* is defined to represent the improvement effect of FRP composite material on the energy dissipation performance of concrete cylindrical members.

(1) $$\lambda =\frac{E_{cu}}{E_{co}}$$

Equation (1): *E_cu_* represents the energy dissipation of the concrete cylindrical member constrained by FRP composite materials, and *E_co_* represents the energy dissipation of unconstrained concrete cylindrical member. In the axial compression test, the energy dissipation coefficient *λ* of the concrete cylindrical member constrained by FRP composite materials is shown in Table 6.

Table 6 shows that that CC-H6 group has the largest energy dissipation coefficient, and that the ductility and seismic performance of this confined concrete cylinder are the best. The control group (CC-FFRP) performed poorly, and the energy dissipation coefficient *λ* was 2.27. However, CC-H5 performs the worst in the reinforcement of hybrid fibers, and the energy dissipation coefficients of CC-H1, CC-H2, CC-H3, CC-H4, CC-H7 and CC-H8 are similar, ranging between those of CC-H5 and CC-H6, which is also consistent with the results obtained in Section 3.2 above.

### 3.4. Stress-Strain Relationship Analysis

**Figure 7:**
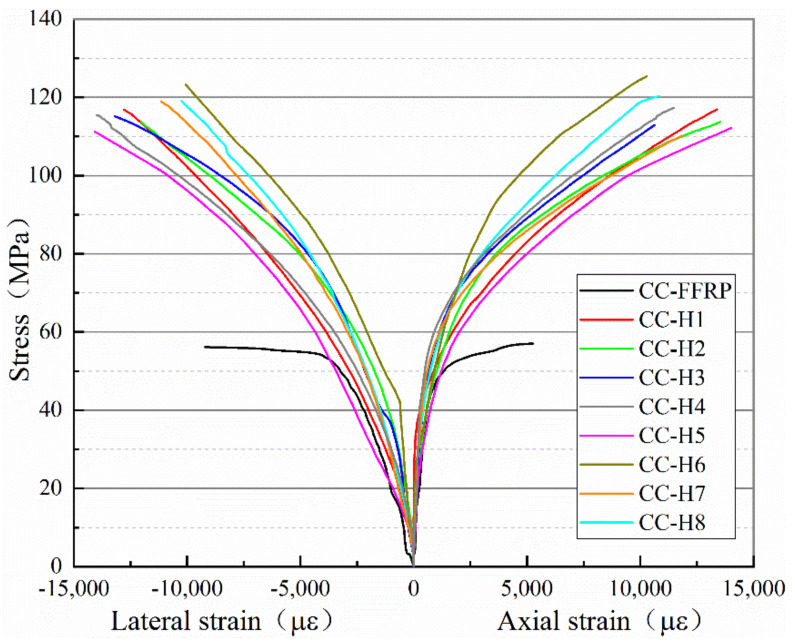
Stress–strain curve relationship of concrete cylinders under unconstrained and different constraint conditions.

When the axial compression test is carried out on the concrete cylindrical member wrapped with FRP composite restraint, the core concrete part is passively restrained. Figure 7 shows the axial compression stress–strain curves of FFRP and various HFRP-confined concrete columns. It is observed that the stress–strain curves of all FRP-confined concrete columns can be divided into two stages. In the first stage, before the applied load reaches the axial compressive strength of the concrete column itself, all curves change very rapidly in the initial stage, which mainly depends on the strength performance of the core concrete [24], which is also consistent with the performance results of the traditional synthetic fiber-confined concrete column. The axial stress of all specimens increases continuously with the applied load and enters the second stage. At this time, FRP constraint stress presents linear growth with the lateral expansion of concrete, showing passive restraint, characterized by the phenomenon of compressive stress rising in the second stage. The slope of each curve in this process is different, and its modulus mainly depends on the performance of each constrained FRP composite [24].

As shown in Figure 7, the axial compressive properties of FFRP and H1-H8 composite-reinforced concrete columns significantly improved. It is undeniable that the ultimate bearing capacity and the circumferential/axial strain of FFRP composite-confined concrete columns are still relatively weak compared with H1-H8 composite-confined concrete columns. The failure trend of stress–circumferential/axial strain curves of HFRP-constrained concrete columns in each group is basically the same, and the main difference is the wrapping sequence of hybrid fiber composites. The strength and ductility of cylindrical concrete specimens are greatly improved by the addition of glass fibers. In comparison, the CC-H5 axial stress–circumferential/axial strain curve has a shallower slope in the second stage, i.e., elastic modulus and maximum failure stress. Except for the CC-H6 and CC-H5 groups, there was no significant difference in other groups as a whole, and its value basically ranged between those of CC-H5 and CC-H6, which was consistent with the conclusions drawn above.

To further research into composite cylinders regarding the strength and ductility increase rate of confined concrete, this paper introduces the improved coefficient of *ω*, its value is being equal to the measured values of the constraints of cylindrical concrete specimens the unconfined concrete column specimens’ measured values divided by the measured values of the unconfined concrete column specimens, and the unit is expressed as a percentage. Then, the effective utilization coefficient k is introduced to express the relationship between the effective circumferential ultimate strain of FRP composite-confined concrete and the ultimate strain of composite performance test, and its magnitude is the ratio of the above two. *f*_cc_′, *ε*_cc_, *ε*_ec_, and *ε*_uc_ represent the ultimate axial stress, the ultimate axial strain, the effective circumferential strain of the FRP composite-confined concrete cylindrical specimens and the ultimate strain value of FRP sheet tensile performance test, respectively. *ω_f_* and *ω_ε_* are the ultimate stress and strain improvement coefficient values of the concrete cylinders constrained by FRP composites, respectively, and the calculation results are shown in Table 7.

As can be seen from Table 7, compared with the unconstrained concrete cylindrical specimens, the stress and axial strain of FFRP composite-confined concrete cylindrical members only increased by 76.9% and 265.5%, while the constraint effect of HFRP composites increased by 247.9%~292.5% and 486.7%~701.0%, respectively. The results show that the restraint effect of HFRP composite is much greater than that of pure flax fiber composite plate, and the strength and ductility of specimens are significantly improved. The axial stress of CC-H6 constrained specimens increased the most, but the axial strain effect was not good, while the effect of CC-H5 was the opposite, which was mainly related to the different fiber layering order. The improvement effect of other HFRP-constrained specimens regarding layering order ranged between those observed for CC-H5 and CC-H6.

The test results also show that the ultimate strain of FRP composites in the tensile performance test is much greater than the effective circumferential fracture strain of its sheet-confined concrete cylinder in the axial compression performance test. The main reasons may be as follows: in the process of specimens subjected to axial compression, the uneven transverse expansion of the internal concrete and the existence of cracks or holes on the surface of the concrete are prone to stress concentration, leading to the local tensile fracturing of FRP composites in advance. When FRP composites are wrapped by bending, their denaturation ability and energy dissipation ductility will be reduced, and there will inevitably be bubbles after manual operation, so this phenomenon will occur.

## 4. Conclusions

In this experiment, the axial compression performance test of HFRP composite-confined concrete cylindrical specimens with the same hybrid ratio is studied. The test design method, test content and loading scheme are introduced. The failure mode, characteristic load, strength and ductility of the compression members and stress-circumferential/axial strain relationship are analyzed and discussed. Based on the results of the study and characterization, the hybrid method is feasible in practical engineering applications, and the following conclusions can be drawn:

(1) The axial compression failure modes of HFRP-confined concrete cylindrical members are basically the same; cracking occurs in the middle part first and spreads to both ends, and the internal core concrete is seriously damaged in a cone shape, which is in obvious contrast to FFRP-confined specimens.
(2) The load–displacement curve of HFRP composite-confined concrete cylinders subjected to axial compression performance test shows that the failure trend is basically consistent throughout the loading process. The ultimate bearing capacity of the CC-H6-confined concrete cylindrical specimen is 91.05% higher than that of the control group (CC-FFRP), and its axial deflection is 11.49% higher than that of the control group. The ultimate bearing capacity of CC-H5 is increased by 84.8%, but the deflection extension is increased by 27.87%. There is no significant difference in the influence of other hybrid layers on the axial compressive performance of concrete columns, and ranged between the values observed for the CC-H6 and CC-H5 groups.
(3) The strength and ductility of concrete columns confined by HFRP can be significantly improved. The CC-H6 group has the largest energy dissipation coefficient (9.79), and its confined concrete columns have the best ductility and seismic performance. CC-H5 performs poorly, and its energy dissipation coefficient is 5.95. The energy dissipation coefficient values of CC-H1, CC-H2, CC-H3, CC-H4, CC-H7 and CC-H8 groups are similar.
(4) The failure trend of the stress–strain curves of FRP-confined concrete cylinders is basically the same, and the main difference is in the failure process of the second stage, which depends on the comprehensive mechanical properties of each HFRP composite. The ultimate stress and elastic modulus of the CC-H6-confined concrete cylinder are obviously higher than other combinations. Further comparison shows that the stress and axial strain of HFFRP composite-confined concrete cylindrical members are increased by 247.9~292.5% and 486.7~701.0%, respectively. The axial stress of CC-H6-constrained specimens increased the most, but the axial strain improvement effect was not good, while the effect of CC-H5 was the opposite, which was mainly related to the different fiber layering sequence. The improvement effect of other HFRP-constrained specimens ranged between that of CC-H5 and CC-H6.

## Figures and Tables

**Table 1:** Mechanical parameters of epoxy resin.

Mechanical Parameters	Tensile Strength (MPa)	Tensile Modulus (MPa)	Bending Strength (MPa)	Elongation (%)
Average value	45.8	2731	70.9	2.04

**Table 2:** Main test instruments and equipment.

Experimental Instrument	Specification and Model	Manufacturer
Universal testing machine	100 kN, WDW-100	Changchun Kexin Test Instrument Co., Ltd., Changchun, China
High speed static strain testing analyzer	16 channel, JM3816A	Yangzhou Jingming Technology Co., Ltd., Yangzhou, China
Pressure sensor	200 t, QLZ-200T	Shenzhen qinheyuan Technology Co., Ltd., Shenzhen, China
Resistance strain gauge	120 Ω, BX120-20AA	Ningbo Yaonan Electrical Equipment Co., Ltd., Ningbo, China

**Table 3:** Mix proportion of concrete per cube (units: kg).

Design Grade	Cement	Water	Fine Aggregate	Coarse Aggregate	Water Reducing Agent
C30	466	605	1174	205	0.9

**Table 4:** Experimental scheme of concrete columns with different composite material constraints.

Concrete Column	Fiber Sheet Wrapping Sequence	Paste Layers	Number of Samples
CC	Unwrapped	0	3
CC-FFRP	F_8_	8	3
CC-H1	F_2_G_2_F_2_G_2_	8	3
CC-H2	G_2_F_2_G_2_F_2_	8	3
CC-H3	FGFGFGFG	8	3
CC-H4	GFGFGFGF	8	3
CC-H5	F_2_G_4_F_2_	8	3
CC-H6	G_2_F_4_G_2_	8	3

**Table 5:** Characteristic loads of concrete columns under different constraints.

Concrete Column	*P_u_* (kN)	Δ*P_u_* (%)	*δ_u_* (mm)	Δ*δ_u_* (%)
CC	253.19	—	6.02	—
CC-FFRP	519.93	105	6.96	15.61
CC-H1	932.95	268	8.11	34.72
CC-H2	901.61	256	7.96	32.23
CC-H3	896.67	254	8.20	36.21
CC-H4	903.51	257	7.99	32.72
CC-H5	745.24	194	8.90	47.84
CC-H6	993.33	292	7.76	28.90

**Table 6:** Energy dissipation coefficients of concrete cylindrical specimens under different constraints.

Concrete Column	Energy Dissipation Coefficient (λ)	Concrete Column	Energy Dissipation Coefficient (λ)
CC-FFRP	2.27	CC-H5	5.95
CC-H1	8.19	CC-H6	9.79
CC-H2	8.37	CC-H7	8.14
CC-H3	8.05	CC-H8	8.64
CC-H4	8.24		

**Table 7:** Comparison of test results of concrete cylinders under different constraints.

Concrete Column	*f_cc_*′ (MPa)	*ε_cc_* (με)	*ε_ec_* (με)	*f_cc_*′/*f_co_*′	*ε_cc_*/*ε_co_*	*ε_ec_*/*ε_uc_*	*ω_f_* (%)	*ω_ε_* (%)
CC-FFRP	66.23	6418	5276	2.05	3.65	0.086	76.9	265.5
CC-H1	118.85	13,391	12,777	3.69	7.63	0.260	262.6	662.6
CC-H2	114.85	13,541	12,035	3.56	7.71	0.245	252.9	671.1
CC-H3	114.23	13,207	10,646	3.54	7.52	0.213	257.3	652.1
CC-H4	115.10	10,836	10,253	3.57	6.17	0.205	273.1	517.1
CC-H5	94.94	14,066	14,010	2.94	8.01	0.274	247.9	701.0
CC-H6	126.54	10,302	10,063	3.92	5.87	0.198	292.5	486.7
CC-H7	113.74	11,851	11,143	3.53	6.75	0.226	252.8	574.9
CC-H8	117.31	11,982	11,493	3.64	6.82	0.236	263.9	582.3

## Data Availability

No new data were created or analyzed in this study. Data sharing is not applicable to this article.
